# Pancreaticoduodenectomy for Ampullary Carcinoma after Clinical Resolution of Necrotizing Pancreatitis with Giant Walled-Off Necrosis: A Case Report

**DOI:** 10.70352/scrj.cr.26-0324

**Published:** 2026-07-01

**Authors:** Kaoru Katano, Takuma Yoshikawa, Yugo Yoshikawa, Yasuharu Kaizaki, Kazuya Maeda

**Affiliations:** 1 Department of Surgery, Fukui Prefectural Hospital, Fukui, Fukui, Japan; 2 Department of Pathology, Fukui Prefectural Hospital, Fukui, Fukui, Japan

**Keywords:** ampullary carcinoma, post-ERCP pancreatitis, walled-off necrosis, colonic perforation, pancreaticoduodenectomy

## Abstract

**INTRODUCTION:**

Ampullary carcinoma is typically treated with pancreaticoduodenectomy (PD). Nevertheless, performing curative resection remains remarkably challenging when the clinical course is complicated by necrotizing pancreatitis and extensive walled-off necrosis (WON). Although resection is occasionally performed after mild pancreatitis, few reports describe curative surgery following severe necrosis requiring necrosectomy. We report a rare case managed with necrosectomy for extensive WON, followed by PD after regression of the necrotic area.

**CASE PRESENTATION:**

A 77-year-old man presented with obstructive jaundice and underwent endoscopic retrograde cholangiopancreatography with biliary metal stent placement and biopsy, leading to a diagnosis of ampullary adenocarcinoma without distant metastasis. Shortly after the procedure, he developed acute necrotizing pancreatitis, which progressed to extensive WON surrounding the pancreatic head and body. Despite conservative treatment, the patient deteriorated due to peritonitis caused by ascending colon perforation adjacent to the necrotic area. Emergency laparotomy revealed widespread peripancreatic necrosis and colonic perforation; right hemicolectomy with ileostomy, open necrosectomy, and multiple drain placements were performed. Over the subsequent 4 months, infection control, rehabilitation, and nutritional support led to gradual regression of the necrosis, marked improvement of inflammatory markers, and no evidence of tumor progression on serial CT. Approximately 6 months after the onset of pancreatitis, a subtotal stomach-preserving PD with lymph node dissection and ileostomy closure was performed. The operation was technically demanding due to dense adhesions and fibrosis, particularly around the superior mesenteric vein. The operative time was 744 min, with an estimated blood loss of 8110 mL. Postoperative complications included a clinically relevant postoperative pancreatic fistula, bile leakage from the hepaticojejunostomy, and stenosis of the portal vein; all were managed conservatively. The patient was discharged on POD 86 and remained alive and recurrence-free 6 months later.

**CONCLUSIONS:**

PD may be feasible in highly selected patients after clinical resolution of necrotizing pancreatitis complicated by extensive WON and colonic perforation. However, surgeons should be aware that the procedure remains highly invasive and technically demanding, even when radiologic and laboratory findings suggest resolution, and should be considered only after thorough multidisciplinary evaluation and careful counselling.

## Abbreviations

ERCP
: endoscopic retrograde cholangiopancreatography
FCSEMS
: fully covered self-expandable metallic stent
PD
: pancreaticoduodenectomy
SMV
: superior mesenteric vein
WON
: walled-off necrosis

## INTRODUCTION

Ampullary carcinoma is an uncommon periampullary malignancy for which PD remains the standard curative treatment. R0 resection is essential for long-term survival, and many patients are diagnosed at a stage where radical surgery is still feasible.^1)^ However, when the clinical course is complicated by severe acute pancreatitis, the indication and timing of PD become highly problematic. In particular, severe pancreatitis with WON can cause extensive local tissue destruction, fibrosis, and adhesions, which may markedly increase the technical difficulty and risk of major upper abdominal surgery.^2)^

Severe pancreatitis with WON is generally managed using a step-up approach, including drainage and, when necessary, surgical necrosectomy.^2)^ In patients who also harbor malignancy in the pancreatic head region, PD may be considered after apparent resolution of pancreatitis, but detailed reports of PD performed after severe pancreatitis progressing to WON are extremely scarce. Even in the available reports, qualitative details on intraoperative adhesions, fibrosis, and the specific technical difficulties of PD in this setting remain limited.^3–5)^ Consequently, there is little practical guidance on whether and when to proceed to PD once these severe pancreatitis-related complications have been brought under control.

Here, we report a case of ampullary carcinoma in which ERCP-induced necrotizing pancreatitis progressed to extensive WON and ascending colonic perforation, requiring right hemicolectomy with ileostomy and surgical necrosectomy, followed months later by PD that achieved R0 resection. We describe the clinical course and operative findings and discuss the feasibility and risks of PD after severe pancreatitis with WON.

## CASE PRESENTATION

A 77-year-old man with a history of hypertension, dyslipidemia, and endovascular aortic repair for an abdominal aortic aneurysm was referred to our hospital for evaluation of obstructive jaundice. Laboratory tests on admission showed elevated hepatobiliary enzymes and cholestasis (total bilirubin 9.4 mg/dL, direct bilirubin 6.4 mg/dL, aspartate aminotransferase 137 IU/L, alanine aminotransferase 231 IU/L, alkaline phosphatase 254 IU/L, γ-glutamyl transferase 1721 IU/L, lactate dehydrogenase 236 IU/L). Contrast-enhanced CT revealed dilatation of the distal common bile duct with a small enhancing lesion at the papilla of Vater, without distant metastasis or major vascular invasion (**Fig. 1A**). ERCP was performed for biliary decompression and diagnostic evaluation. Biliary cannulation was achieved using the pancreatic guidewire–assisted technique, with a cannulation time of 13 min and a total procedure time of 78 min. Minimal pancreatic duct opacification occurred during the procedure. Cholangiography showed biliary dilatation with a filling defect in the distal bile duct near the papilla (**Fig. 1B**). A FCSEMS, HANAROSTENT Multi-Hole (M.I.Tech, Pyeongtaek, Korea) measuring 10 mm in diameter and 5 cm in length, was placed across the papilla, and adequate bile drainage was confirmed. Because pancreatic duct manipulation had occurred, rectal diclofenac 50 mg and additional intravenous hydration were administered after ERCP. No immediate procedural complication was observed. Biopsy of the papillary lesion revealed adenocarcinoma, leading to a diagnosis of ampullary carcinoma.

**Fig. 1:**
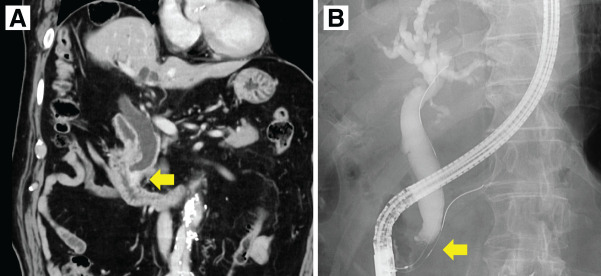
Initial imaging at diagnosis of ampullary carcinoma. (**A**) Contrast-enhanced CT showing dilatation of the distal common bile duct with a small enhancing lesion at the papilla of Vater (arrow), without distant metastasis or major vascular invasion. (**B**) ERCP demonstrating a filling defect extending from the distal bile duct to the papilla (arrow). ERCP, endoscopic retrograde cholangiopancreatography

Shortly after ERCP, the patient developed acute pancreatitis with new-onset epigastric pain, and the serum amylase level increased to 753 IU/L. Contrast-enhanced CT performed 4 days after ERCP demonstrated diffuse enlargement of the pancreas with extensive peripancreatic fat stranding and non-enhancing areas in the pancreatic head and body, consistent with necrotizing pancreatitis with large peripancreatic collections (**Fig. 2A**). There was no organ failure in the respiratory, cardiovascular, or renal systems, but substantial peripancreatic fluid collections were present as a local complication. According to the revised Atlanta classification, this episode was categorized as moderately severe acute pancreatitis.

**Fig. 2:**
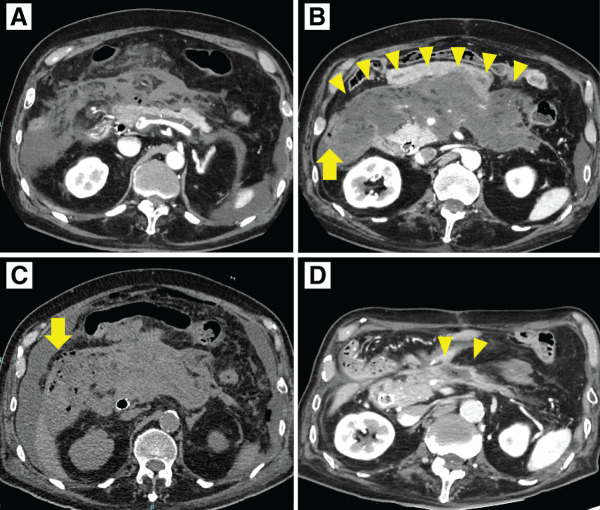
Serial CT images showing the evolution and resolution of necrotizing pancreatitis with WON. (**A**) Four days after ERCP, diffuse enlargement of the pancreas with extensive peripancreatic fat stranding and non-enhancing areas in the pancreatic head and body, consistent with acute necrotizing pancreatitis and large peripancreatic collections. (**B**) Approximately 30 days after ERCP, maturation of these collections into a large WON surrounding the pancreas (arrowheads) and extending along the ascending colon (arrow). (**C**) At clinical deterioration, marked enlargement of the WON with internal gas and evidence of perforation of the ascending colon adjacent to the collection, accompanied by free intraperitoneal air (arrow). (**D**) About 4 months after open necrosectomy and drainage, near-complete regression of the WON with only small residual peripancreatic collections and scarring (arrowheads). ERCP, endoscopic retrograde cholangiopancreatography; WON, walled-off necrosis

Despite intensive conservative management, these collections gradually evolved into WON. CT performed approximately 30 days after ERCP showed maturation of the collections into a large WON surrounding the pancreas and extending along the ascending colon (**Fig. 2B**). About 40 days after ERCP, the patient developed worsening abdominal pain, fever, and signs of peritonitis. CT revealed marked enlargement of the WON with air bubbles and evidence of perforation of the ascending colon adjacent to the collection, accompanied by free intraperitoneal air (**Fig. 2C**). Emergency laparotomy was performed. Intraoperatively, a large necrotic cavity containing turbid fluid and debris was found around the pancreas, in continuity with a perforation of the ascending colon. The patient underwent right hemicolectomy with end ileostomy, surgical necrosectomy of the peripancreatic necrotic tissue, and placement of multiple drains for continuous external drainage (**Fig. 3**). Postoperatively, he was managed in the ICU. Under broad-spectrum antibiotics, nutritional support, and careful drain management, his general condition gradually improved.

**Fig. 3:**
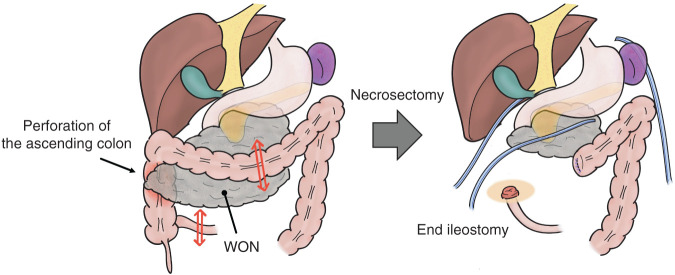
Schematic illustration of emergency surgery for giant WON with colonic perforation. Surgical schema showing a giant WON occupying the peripancreatic and right paracolic regions, causing perforation of the ascending colon. A right hemicolectomy with end ileostomy, open necrosectomy of the peripancreatic necrotic tissue, and placement of multiple drains for continuous external drainage were performed. WON, walled-off necrosis

Over the following months, serial CT scans demonstrated progressive shrinkage of the peripancreatic collections and maturation of the cavity walls, and drain output decreased. He was discharged home with 1 drain left in place on POD 72 and continued convalescence as an outpatient. CT performed about 5 months after ERCP showed that the WON had almost completely regressed, with only small residual collections (**Fig. 2D**). Serum C-reactive protein gradually decreased to near-normal levels (1.31 mg/dL). The patient’s nutritional status also improved with nutritional support, with serum albumin increasing from 1.2 g/dL before emergency surgery to 2.6 g/dL by this time. During this period, there was no radiologic evidence of distant metastasis or clear progression of the ampullary tumor. Serum tumor markers showed an elevated CA19-9 level of 237.6 U/mL, whereas CEA remained within the normal range. Based on these findings, we judged that pancreatitis- and WON-related inflammation had sufficiently subsided and that the patient’s general condition was acceptable for curative PD despite residual nutritional risk. The chronological sequence of clinical events, interventions, imaging findings, and inflammatory marker trends is summarized in **Fig. 4**.

**Fig. 4:**
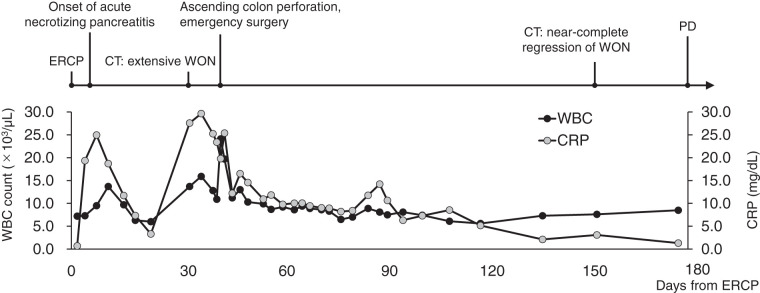
Clinical course from ERCP to PD. The figure summarizes the major clinical events and trends in WBC count and CRP level from ERCP to PD. WBC count and CRP initially improved after conservative management, but increased again after the development of WON. Serum CRP subsequently decreased gradually during the recovery period and reached a near-normal level before PD. CRP, C-reactive protein; ERCP, endoscopic retrograde cholangiopancreatography; PD, pancreaticoduodenectomy; WBC, white blood cell; WON, walled-off necrosis

After thorough preoperative counselling of the patient and his family regarding the high operative risk and the possibility that the procedure might have to be abandoned intraoperatively if deemed unresectable or excessively hazardous, PD was performed approximately 6 months after the onset of pancreatitis. At laparotomy, the upper abdomen showed dense adhesions and severe fibrosis involving the pancreas, gastric antrum, duodenum, and surrounding tissues (**Fig. 5A**). In contrast, adhesions and scarring around the hepatoduodenal ligament were only mild. After Kocher’s maneuver, the stomach was divided at the antrum, followed by lymphadenectomy along the hepatoduodenal ligament and cholecystectomy. Accessing the pancreatic head required meticulous stepwise adhesiolysis. The SMV was encased in dense scar tissue, which made secure encirclement and taping difficult. However, a narrow but safe dissection plane could be developed between the anterior surface of the SMV and the posterior aspect of the pancreatic neck, allowing taping of the pancreatic parenchyma (**Fig. 5B**). Once this plane had been secured, we judged that pancreatic head resection was feasible, and the pancreas was transected at this level (**Fig. 5C**). The common hepatic duct was then divided. Because of severe fibrosis and tissue induration, separation of the pancreatic head from the SMV was extremely difficult, and an iatrogenic injury to the SMV wall occurred due to the dense fibrosis; this was successfully repaired using 6-0 polypropylene sutures (Prolene; Ethicon, Raritan, NJ, USA). By carefully dissecting through the dense scar tissue, the pancreatic head was eventually separated from the SMV, and standard subtotal stomach-preserving PD with regional lymph node dissection was completed. Reconstruction was performed by pancreaticojejunostomy using the Blumgart duct-to-mucosa technique, hepaticojejunostomy, and gastrojejunostomy. At the same time, the pre-existing ileostomy was closed, and bowel continuity was restored by ileotransverse anastomosis. The total operative time was 744 min, and the estimated blood loss was 8110 mL. The patient required transfusion of 20 units of red blood cells, 22 units of fresh frozen plasma, and 20 units of platelet concentrate.

**Fig. 5:**
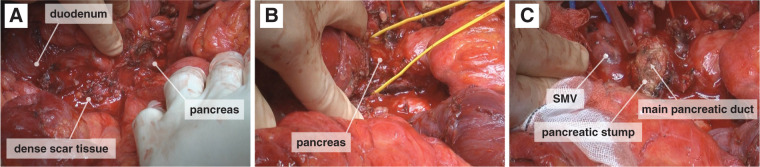
Intraoperative findings during PD. (**A**) Laparotomy view showing dense adhesions and severe fibrosis in the upper abdomen involving the pancreas, gastric antrum, duodenum, and surrounding tissues. (**B**) A narrow but safe dissection plane was developed between the anterior surface of the SMV and the posterior aspect of the pancreatic neck, allowing taping of the pancreatic parenchyma. (**C**) The pancreatic parenchyma was transected anterior to the SMV. PD, pancreaticoduodenectomy; SMV, superior mesenteric vein

Histopathological examination of the resected specimen revealed a well to moderately differentiated adenocarcinoma of the ampulla of Vater, measuring 23 mm in greatest diameter (**Fig. 6A**). The tumor invaded the pancreatic parenchyma and duodenal wall. No lymph node metastasis was identified. The final pathological stage was pT3N0M0 according to the 8th edition of the Union for International Cancer Control classification, and all resection margins were negative (R0 resection). In the surrounding pancreatic and peripancreatic tissues, marked fibrosis and scarring were observed, and residual necrotic tissue adjacent to the pancreatic parenchyma was also identified (**Fig. 6B**).

**Fig. 6:**
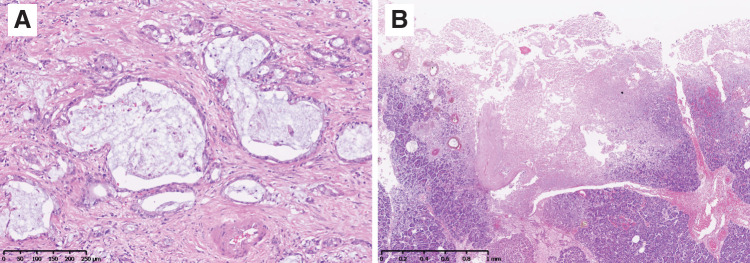
Histopathological findings of the resected specimen. (**A**) Well to moderately differentiated adenocarcinoma of the ampulla of Vater (hematoxylin and eosin staining). (**B**) Fibrosis and scarring in the surrounding pancreatic and peripancreatic tissues, with residual necrotic tissue adjacent to the pancreatic parenchyma (hematoxylin and eosin staining).

The postoperative course was complicated by a clinically relevant postoperative pancreatic fistula (International Study Group on Pancreatic Surgery grade B) and bile leakage from the hepaticojejunostomy. Both were managed conservatively with prolonged drainage and nutritional support. Imaging also revealed portal vein stenosis or occlusion; however, the patient did not develop clinical liver failure. After removal of all drains, he was discharged from the hospital on POD 86. At the latest follow-up, 6 months after surgery, he remained alive without evidence of recurrence under ongoing surveillance.

## DISCUSSION

This case provided 2 important clinical suggestions. First, it shows that curative PD may still be considered even after the development of extensive WON. Previous studies have reported that PD after acute pancreatitis carries an increased risk of operative difficulty and postoperative morbidity.^3,4,6)^ For malignant diseases such as pancreatic cancer, several authors have suggested that surgery should ideally be delayed until inflammation has clearly subsided and local tissue conditions have stabilized, typically at least 3–4 weeks after the onset of pancreatitis, when oncologically acceptable.^7)^ Asari et al. further proposed that an interval of at least 3 months may be required after severe acute pancreatitis to allow peripancreatic inflammation to settle.^8)^ However, among the literature we reviewed, reports in which PD was performed for malignancy after the development of WON severe enough to require necrosectomy were extremely limited. To our knowledge, the case described by Moyon et al.,^9)^ in which necrotizing pancreatitis with WON due to ampullary carcinoma was treated by minimally invasive necrosectomy followed by delayed pylorus-preserving PD, is the only clearly comparable report, and even in that case, detailed information on intraoperative difficulty is sparse. For WON itself, a step-up approach, including endoscopic or percutaneous drainage and, if necessary, necrosectomy, has been widely advocated as standard management.^10,11)^ In our patient, ascending colonic perforation secondary to extensive WON necessitated right hemicolectomy, diverting ileostomy, and open necrosectomy, followed by approximately 4 months of drainage, rehabilitation, and nutritional support until inflammation had subsided. Under these conditions, we proceeded to PD with curative intent. This experience suggests that with appropriate stepwise management, even very extensive WON can, in selected patients, be controlled sufficiently for PD to be attempted as radical surgery.

Second, although preoperative findings suggested near-complete resolution of pancreatitis and WON, the operative field remained extremely hostile. After diverting ileostomy, necrosectomy, and prolonged drainage, cross-sectional imaging demonstrated marked improvement of peripancreatic collections, and inflammatory markers had markedly improved, indicating apparent healing of pancreatitis. Nevertheless, at laparotomy the upper abdomen was heavily scarred, with dense adhesions around the pancreas, duodenum, and transverse colon. Although a prolonged and technically demanding operation had been anticipated, the actual blood loss of 8110 mL exceeded our preoperative expectation. This massive blood loss was mainly attributable to diffuse oozing from severely inflamed and fibrotic tissues and bleeding during dissection around major vessels, including the SMV. These observations indicate that even when WON appears to have largely resolved radiologically, substantial fibrosis and scarring may persist and create a severely hostile operative field. Thus, PD after WON is technically feasible but carries considerable risk, which must not be underestimated based solely on favorable imaging and laboratory findings.

Post-ERCP pancreatitis occurs in approximately 5%–10% of procedures,^12,13)^ and a subset of these patients develop severe acute pancreatitis. Among patients with severe acute pancreatitis, WON has been reported to occur in about 30%.^14)^ In a multicenter study, Ikarashi et al.^14)^ found that high BMI, disseminated intravascular coagulation, and post-ERCP pancreatitis were independent risk factors for the development of WON. Thus, severe pancreatitis with WON, including cases triggered by ERCP, appears to be uncommon but not truly exceptional in biliary and periampullary practice. It is therefore possible that some patients with potentially resectable malignancy are not ultimately offered curative surgery because of concerns about operative risk. In principle, it seems reasonable to defer surgery until inflammatory changes have clearly subsided. However, there is no consensus regarding how long surgeons should wait after severe pancreatitis or WON, and prolonged postponement raises the opposing concern of tumor progression. Although ampullary carcinoma generally has a more favorable prognosis than pancreatic ductal adenocarcinoma, it remains a malignant disease with potential for local progression, lymph node metastasis, and distant spread. Therefore, prolonged deferral of surgery for resectable ampullary carcinoma cannot be regarded as oncologically ideal, even when required for control of pancreatitis-related inflammation. Accordingly, the optimal timing of PD remains difficult to determine and requires balancing adequate control of pancreatitis-related complications against the risk of losing the opportunity for curative resection. In the present case, there was no evidence of distant metastasis or obvious tumor progression during the waiting period, allowing surgery to be deferred for approximately 6 months until local inflammation had subsided and the patient’s general condition had recovered sufficiently. Although the operation involved prolonged operative time and massive blood loss, the patient ultimately recovered without fatal complications. Thus, the timing of surgery in this case was considered acceptable, although not oncologically ideal, in balancing operative risk against the chance of curative resection.

The present case also raises an important issue regarding the initial biliary drainage strategy. Although rectal diclofenac and additional intravenous hydration were administered after ERCP, pancreatic duct manipulation was performed, including pancreatic guidewire-assisted cannulation and minimal pancreatic duct opacification. These factors may have contributed to the development of post-ERCP pancreatitis. In addition, the FCSEMS was placed across the papilla to obtain reliable biliary drainage before definitive surgery. However, in patients with periampullary tumors, an FCSEMS placed across the papilla may cause mechanical compression or functional obstruction of the pancreatic duct orifice and thereby contribute to post-ERCP pancreatitis. In retrospect, because the tumor was considered resectable and neoadjuvant therapy was not planned, placement of a plastic stent may also have been a reasonable option. After the onset of pancreatitis, early removal of the FCSEMS, conversion to endoscopic nasobiliary drainage, or replacement with a plastic stent might also have been considered, although whether such intervention would have altered the subsequent clinical course remains uncertain.

The timing of intervention for WON is another important issue. In the present case, inflammatory markers initially improved with conservative management after the onset of pancreatitis, but later increased again after the development of extensive WON (**Fig. 4**). Body temperature transiently increased to 38.1°C at the onset of pancreatitis but remained mostly below 37.5°C thereafter until emergency surgery. Although there was no sustained high fever or definitive evidence of infected necrosis or sepsis requiring emergency intervention, the patient had extensive WON with persistent inflammatory activity. In retrospect, intervention for WON may have been delayed in this case. Earlier drainage or necrosectomy might have prevented ascending colonic perforation, reduced the local inflammatory burden, and potentially decreased the severity of fibrosis and adhesions encountered during subsequent PD. Recent studies have suggested that earlier endoscopic intervention may accelerate clinical improvement in selected patients with WON.^15)^ However, whether earlier intervention would have altered the clinical course in the present case remains uncertain.

Our case, in which R0 resection was fortunately achieved, should not be generalized to all patients with periampullary malignancy after severe pancreatitis. We did not apply any objective scoring system to quantify adhesion severity or fibrosis, and intraoperative decisions were based on the subjective judgment of an experienced hepato-biliary-pancreatic surgeon. Furthermore, the long-term oncologic outcome of this patient remains to be clarified with additional follow-up, and we cannot determine whether the potential survival benefit of R0 resection will always justify the high operative risk in similar cases. Referral to a high-volume pancreatic surgery center was not actively pursued because our team was familiar with the patient’s entire clinical course, including the initial emergency surgery and intra-abdominal findings. However, consultation with, or referral to, a specialized center may also be considered in similar cases. In retrospect, omission of simultaneous ileostomy closure may have reduced operative time and surgical stress. In addition, earlier vascular control or collaborative planning with vascular surgeons may be useful when dense perivascular fibrosis, particularly around the SMV, is anticipated. Staged reconstruction, including delayed pancreatojejunostomy, might also be considered in selected extremely high-risk cases, although evidence for such strategies remains limited. These risks must be clearly discussed with patients and their families during preoperative counselling. Given the unpredictability of local tissue conditions, surgeons must be prepared for the possibility that PD will prove infeasible or excessively hazardous at laparotomy and that resection must be abandoned or converted to a less radical or staged procedure.

## CONCLUSIONS

This case demonstrates that PD can achieve curative resection in a patient with ampullary carcinoma after severe pancreatitis with WON and colonic perforation, but at the cost of extreme operative difficulty and invasiveness. When considering PD in this setting, careful patient selection, clear criteria for the timing of surgery, and thorough preoperative counselling are essential. Further case accumulation is needed to establish more robust guidance on indication, timing, and operative strategy in this challenging clinical context.

## DECLARATIONS

### Availability of data and materials

The data supporting the findings of this case report are available from the corresponding author upon reasonable request.
